# Direct Anthropometry Overestimates Cranial Asymmetry—3D Digital Photography Proves to Be a Reliable Alternative

**DOI:** 10.3390/diagnostics13101707

**Published:** 2023-05-11

**Authors:** Felix Nieberle, Steffen Spoerl, Lisa-Marie Lottner, Gerrit Spanier, Johannes G. Schuderer, Mathias Fiedler, Michael Maurer, Nils Ludwig, Johannes K. Meier, Tobias Ettl, Torsten E. Reichert, Juergen Taxis

**Affiliations:** Department of Cranio- and Maxillofacial Surgery, Hospital of the University of Regensburg, Franz-Josef-Strauß-Allee 11, 93053 Regensburg, Germany; felix.nieberle@stud.uni-regensburg.de (F.N.); steffen.spoerl@ukr.de (S.S.); lisamarielottner@gmail.com (L.-M.L.); gerrit.spanier@ukr.de (G.S.); johannes.schuderer@ukr.de (J.G.S.); mathias1.fiedler@ukr.de (M.F.); michael.maurer@ukr.de (M.M.); nils.ludwig@ukr.de (N.L.); johannes.meier@ukr.de (J.K.M.); tobias.ettl@ukr.de (T.E.); torsten.reichert@ukr.de (T.E.R.)

**Keywords:** positional head deformation, deformational plagiocephaly, deformational brachycephaly, three-dimensional photography, orthotic helmet therapy

## Abstract

This study compared manual and digital measurements of plagiocephaly and brachycephaly in infants and evaluated whether three-dimensional (3D) digital photography measurements can be used as a superior alternative in everyday clinical practice. A total of 111 infants (103 with plagiocephalus and 8 with brachycephalus) were included in this study. Head circumference, length and width, bilateral diagonal head length, and bilateral distance from the glabella to the tragus were assessed by manual assessment (tape measure and anthropometric head calipers) and 3D photographs. Subsequently, the cranial index (CI) and cranial vault asymmetry index (CVAI) were calculated. Measured cranial parameters and CVAI were significantly more precise using 3D digital photography. Manually acquired cranial vault symmetry parameters were at least 5 mm lower than digital measurements. Differences in CI between the two measuring methods did not reach significance, whereas the calculated CVAI showed a 0.74-fold decrease using 3D digital photography and was highly significant (*p* < 0.001). Using the manual method, CVAI calculations overestimated asymmetry, and cranial vault symmetry parameters were measured too low, contributing to a misrepresentation of the actual anatomical situation. Considering consequential errors in therapy choices, we suggest implementing 3D photography as the primary tool for diagnosing deformational plagiocephaly and positional head deformations.

## 1. Introduction

“Back to Sleep,” a campaign launched in April 1992 by the American Academy of Pediatrics (AAP), was geared towards preventing sudden infant death syndrome (SIDS) [1]. By discouraging parents from letting their children sleep face down or on their sides, as prone and side-lying sleeping positions were associated with a higher incidence of SIDS, the AAP successfully contributed to more than a 50% decrease in the SIDS rate in the US [2,3,4]. Even though this remarkable reduction in child mortality was achieved, the incidence of head-shape abnormalities went up simultaneously with the increased time babies spent on their backs [5,6,7,8,9]. Some studies mentioned increases of up to 600% [10]. Mawji et al., among others, found a certain degree of plagiocephaly in almost half of the infants under three months of age [4,9,10,11]. Those head deformities were directly related to changes in sleeping positions, resulting in deformational plagiocephaly (DP) [4,5,12].

DP can be defined as an asymmetric skull deformity presenting a unilateral flattening of the occiput and no association with craniosynostosis [4,5,11,12,13,14]. Furthermore, DP is often accompanied by an ipsilateral protrusion of the forehead and an anterior shift of the ipsilateral ear, which can be recognized in comparison to the position of the other ear as an ear offset (EO) [14,15].

The pathophysiology behind this deformity results from constant external forces acting on the child’s skull at the area of contact with the surface it is lying on. The head exerts a force on the resting surface equal to its weight multiplied by the force of gravity, and in line with Newton’s first law, the mattress creates an equal but opposing counterforce to the infant’s head. That force hinders growth in this specific area. Therefore, ongoing volume increases are diverted to areas with lower counterforces—more precisely, the ipsilateral anterior and contralateral posterior regions of the skull [11,16]. At last, this compensatory growth results in cranial deformation. Thus, the elaborated pathomechanism is why head shapes are symmetrical at birth, and then, around seven weeks after delivery, parents start recognizing deformations of their infants’ heads [16,17]. 

Further, identifying risk factors predisposing neonates to plagiocephaly is critical [13,18]. Those include, among others, premature birth, a restrictive intrauterine environment, delivery by forceps or vacuum extraction, male gender, congenital muscular torticollis, and a supine sleeping position [4,5,12,17,18]. Furthermore, nutrition during pregnancy and childhood could be identified as risk factors, as the inadequate consumption of vitamin D and folic acid was associated with cranial deformations in the infant [13].

Plagiocephaly is typically diagnosed through a physical examination by the child’s family doctor or pediatrician. Herein, another result of the Back to Sleep campaign could be recognized. The average age of children referred for evaluation of DP and craniosynostosis decreased after 1992, representing an increased awareness of this malady [6,8,13]. If the diagnosis is still unclear, the infant is referred to a specialist for further testing, such as ultrasound, magnetic resonance imaging, or computed tomography of the head to rule out craniosynostosis [4,15,16]. 

Surgical treatment is rarely necessary for infants with DP, but they often require a conservative treatment plan [10]. In 2004, Argenta et al. introduced a clinical classification to help quantify the degree of deformity and guide the selection of the appropriate treatment [15]. However, it is still difficult to determine whether a patient requires orthotic helmet treatment, physiotherapy, positional treatment, or no treatment at all due to inaccurate cranial deformity measurements and the lack of clear guidelines for a standardized diagnostic method [4,19,20,21]. 

Measuring with calipers as the common anthropometric tool is the simplest and cheapest way to assess the severity of plagiocephaly during physical examination [4,19,22,23]. Although reasonably precise regarding intra-rater reliability, Mortenson et al. discovered insufficient inter-rater reliability in caliper measurements. Subsequently, they recommended further development in this area of diagnosis to measure the degree of plagiocephaly more accurately [20]. Additionally, manual caliper measurements rely on the cooperation of the infants, which is sometimes low. Constant moving compromises the measurement and makes it challenging and cumbersome to locate the bony landmarks complex, ultimately leading to imprecise values [19,20,23,24].

First, attempts to simplify and objectify measuring the degree of plagiocephaly by photographic imaging, even though arbitrary and prone to some errors, were made in 1981 by Clarren et al. [25]. In 2004, Zonenshayn et al. created an objective semiautomated measurement technique of plagiocephaly by photographic imaging from a vertex view with an elastic band placed around the infant’s head [24]. Still, in those two-dimensional (2D) imaging methods, a standard head position of the examinee is mandatory to minimize parallax and to find the ideal photographic line-of-sight perpendicular to the plane of the greatest circumference [24]. The mentioned requirements are often challenging to meet in clinical practice, and 2D photography is still incapable of fully capturing the three-dimensional (3D) complexity of the infant’s head [19,24,26,27,28,29]. 

In summary, no standardized diagnostic methods exist for accurately assessing cranial asymmetry in children with DP in every clinical setting without too much dependence on the examiner’s experience or the examinee’s cooperation. Further, direct anthropometry has some issues with the reliability and reproducibility of measurements, which makes it less objective and comparatively unsuitable for initial diagnostic purposes. Additionally, it seems that 2D imaging techniques are unfit to capture all the 3D features of a human head with the necessary level of precision and detail. Consequently, this study aimed to evaluate digital 3D photographic imaging as an objective, sufficiently precise, and reproducible alternative to manual anthropometric measurements in children with DP. 

## 2. Materials and Methods

### 2.1. Patient Selection and Data Collection

This retrospective monocentric study was performed at the Department of Cranio- and Maxillofacial Surgery, University Hospital Regensburg, Germany. A total of 111 infants with non-synostotic plagiocephaly (*n* = 103) and brachycephaly (*n* = 8) undergoing orthotic helmet therapy were included. The recruitment period spanned five years, from 2016 to 2020. The following clinical parameters were assessed: sex, age, age at the start of therapy in months, diagnosis (plagiocephaly and/or brachycephaly), skull base involvement, affected side, therapy duration in months, type of birth (spontaneous birth, spontaneous delivery with suction cup, cesarean section, or emergency section), and multiple births.

### 2.2. Manual and Three-Dimensional Measurement

**Figure 1:**
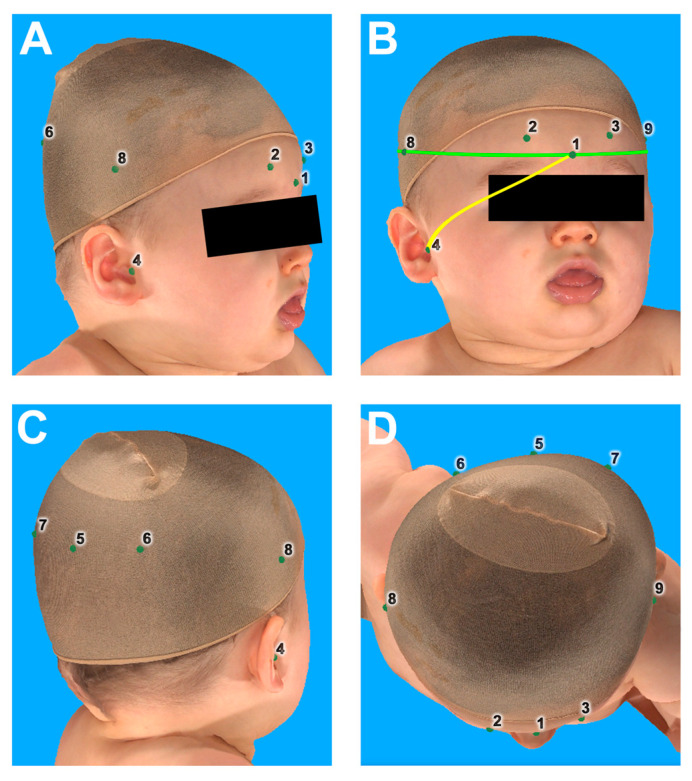
Landmarks set within the software for measuring the individual parameters with the representation of the head from the right (**A**), front (**B**), back (**C**), and top (**D**). Green line = head circumference; yellow line = distance glabella to tragus right; 1 = glabella; 2 = most prominent point supraorbital right at a 30-degree angle to the median plane; 3 = most prominent point supraorbital left at a 30-degree angle to median plane; 4 = tragus right, 5 = widest point of the head occipital; 6 = most deficient point occipital at 30 degrees to the median plane; 7 = most prominent point occipital at 30 degrees to the median plane; 8 = widest point of head lateral right; 9 = widest point of head lateral left.

The head was measured by two independent examiners (J.T. and L.L.) using a tape measure and a commercial anthropometric head caliper. Then, an average value was calculated from the two manual measurements. A comparative 3D digital photography was performed at a maximum interval of 14 days after the manual measurement using a five-camera system and multi-flash lighting system (Vectra M5 3D Imaging System, Canfield Scientific, Parsippany, NJ, USA) to keep growth-related differences at a minimum. After previous calibration of the camera system according to the manufacturer’s guidelines, a fixed toddler chair was placed in the center of the camera setup. Two cameras were positioned in front of the chair and two behind the chair, each at an angle of 45 degrees (Figure S1). The fifth camera was positioned directly above. Photographs were taken of the examinee sitting upright in the chair and facing forward. Using the Vectra Analysis Module software (Canfield Scientific, Parsippany, NJ, USA), digital measurement points were first generated on the 3D models of the infant head obtained in this way, analogous to the manual measurement points. Subsequently, the parameters mentioned below were again measured by two independent examiners (J.T. and L.L.) using the integrated measuring tool, and average values were finally calculated. Infants were wearing single-use silk stockings over their heads to eliminate disruptive factors such as hair for the most accurate acquisition of their head profile. Manually and digitally collected values included: head circumference, head length, head width, right (RD) as well as left (LD) diagonal head length (at a 30° angle to the median sagittal plane), difference between the two diagonals and the distance of the glabella to the right and left tragus (GTR and GTL, respectively) as a reference to skull base involvement. Figure 1 shows a representative photograph with all reference points.

### 2.3. Data Analysis

In line with the suggestions by Loveday et al. [30] and Mortenson et al. [20], the cranial index (CI) and the cranial vault asymmetry index (CVAI) were obtained. CI was calculated by dividing the cranial width by the cranial length.

$$\mathrm{Cranial}\;\mathrm{Index}\; [\%]=\frac{\mathrm{cranial}\;\mathrm{width}\;}{\mathrm{cranial}\;\mathrm{length}\;}\times 100$$

CVAI was calculated by the difference in length between the two diagonal lines (A and B, where A > B) drawn 30° from the median plane, divided by the shorter diagonal line (diagonal B). A CVAI > 3.5% is considered asymmetric and, therefore, pathological [30].

$$\mathrm{Cranial}\;\mathrm{Vault}\;\mathrm{Asymmetry}\;\mathrm{Index}\; [\%]=\frac{\;\mathrm{diagonal}\;A-\mathrm{diagonal}\;B}{\mathrm{diagonal}\;B}\times 100$$

3D digital data were analyzed using Vectra Analysis Module software (Canfield Scientific, Parsippany, NJ, USA).

### 2.4. Statistical Analysis

The statistical analysis was performed using IBM SPSS Statistics 26.0 (IBM Corp., Armonk, NY, USA) and GraphPad Prism 9.0 (GraphPad Software, La Jolla, CA, USA). The mean (MV), standard deviation (SD), and standard error of the mean (SEM) of the manual and 3D measurements were calculated. A paired Mann–Whitney U test was used to analyze the differences between the manual and 3D-based approaches. The significance level was defined as *p* < 0.05.

## 3. Results

### 3.1. Clinicopathological Characteristics

Between 2016 and 2020, 111 infants were included in the present study. From this cohort, 41 were female and 70 were male infants undergoing orthotic helmet therapy in our hospital, with an average age of 5.59 ± 1.76 months at the beginning of the treatment, ranging from 3 to 12 months. In 103 (92.8%) cases, plagiocephaly was the primary diagnosis. The remaining 8 (7.2%) infants were presented with brachycephaly. A total of 101 (91%) infants were diagnosed with skull base involvement, while the other 10 (9%) infants did not show signs of skull base involvement. Out of the 111 cases, 65 (58.6%) showed a right-accented flattening of the occiput. Left-accented flattening was noticed in the other 46 (41.4%) infants. The mean duration of treatment was 150.78 ± 39.11 days, ranging from 58 to 266 days. With regard to the delivery, 61 (55%) infants had a spontaneous birth, while 7 (6.3%) had a spontaneous delivery with a suction cup. A total of 39 infants (35.1%) were born by cesarean section, and 4 were born by emergency section (3.6%). A total of 11 infants (9.9%) in the cohort were twins (Table 1).

### 3.2. Head Measurements Using Manual and 3D-Based Measurement Methods

In the manual method, head circumference was measured using a tape measure. A mean value of 42.76 ± 1.87 cm was acquired. The mean head length was 13.53 ± 0.79 cm, and the mean head width was 12.10 ± 0.77 cm, measured by using anthropometric head calipers. RD and LD were also generated by using the calipers, with mean values of 13.64 ± 1.06 cm and 13.41 ± 1.12 cm, respectively. From those diagonal measurements, a diagonal difference was calculated with a mean value of 1.44 ± 0.41 cm. Additionally, the distance from the glabella to the tragus on both sides was measured. GTR was determined at a mean value of 11.26 ± 0.94 cm, and GTL was determined at a mean value of 11.35 ± 0.93 cm. From those measurements, the CI and CVAI were calculated, with an average of 89.67 ± 7.28% and 11.33 ± 3.56%, respectively (Table 2).

**Figure 2:**
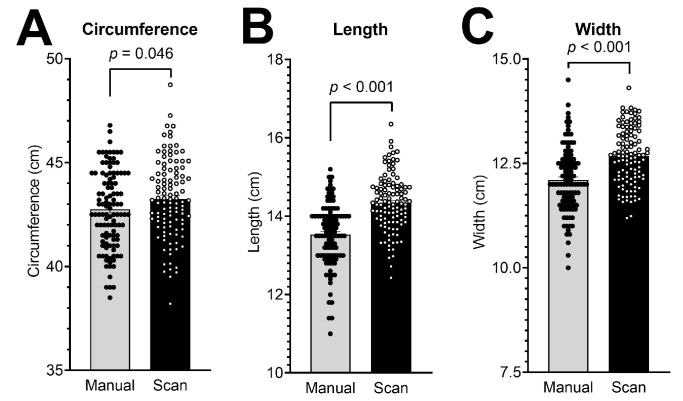
Measured cranial parameters by manual assessment (tape measure and anthropometric head calipers) and 3D photographs (scan) in 111 infants. (**A**) Head circumference, (**B**) head length, and (**C**) head width. Bars represent means ± SEM.

Through 3D digital photography, the same head parameters were assessed. The head circumference showed a mean value of 43.24 ± 1.90 cm, which represents a 1.01-fold increase over the manual method (*p* = 0.046; Table 2; Figure 2A). Further, 1.06- and 1.05-fold increases over the caliper measurements were observed in the respective digital measurements of the head length and head width, with mean values of 14.33 ± 0.72 cm and 12.67 ± 0.68 cm, respectively (both *p* < 0.001; Table 2; Figure 2B,C). 

**Figure 3:**
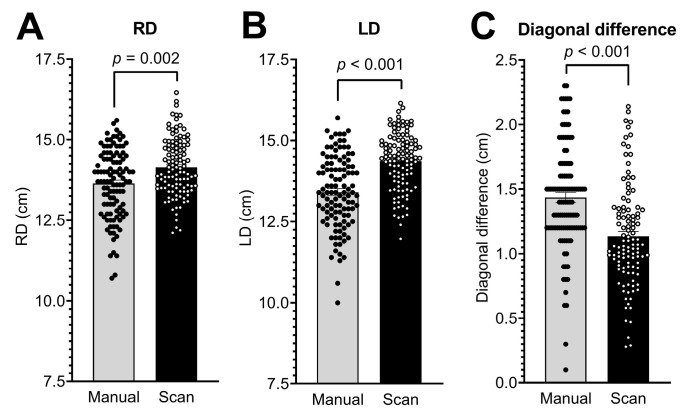
Measurement of (**A**) right diagonal head length (RD); (**B**) left diagonal head length (LD); and (**C**) difference between the two diagonals by manual assessment and 3D photographs (scan) in 111 infants. Bars represent means ± SEM.

Additionally, increased values of RD and LD were recorded in the 3D photographs, with an RD of 14.15 ± 0.90 cm and an LD of 14.37 ± 0.89 cm. Compared to the manual measurement method, this was a 1.04- and 1.07-fold increase, respectively (*p* = 0.002 and *p* < 0.001; Table 2; Figure 3A,B). In contrast to these increased values by using the digital method, we observed a 0.78-fold decrease in diagonal difference when using 3D photography, with a mean value of 1.13 ± 0.39 cm (*p* < 0.001; Table 2; Figure 3C). 

**Figure 4:**
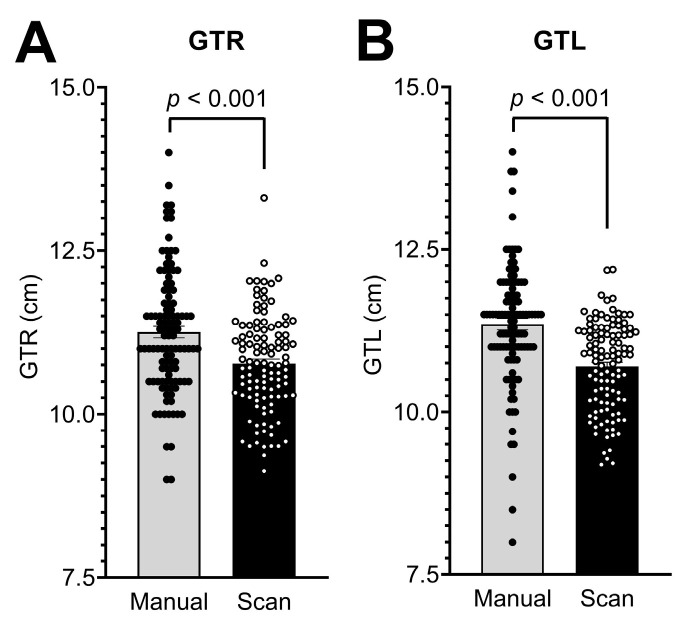
Measurement of (**A**) the distance of the glabella to the right tragus (GTR) and (**B**) the distance of the glabella to the left tragus (GTL) by manual assessment and 3D photographs (scan) in 111 infants. Bars represent means ± SEM.

At 10.77 ± 0.75 cm, a 0.96-fold decrease in GTR measurement was recorded digitally, and at 10.70 ± 0.68 cm, a 0.94-fold reduction in GTL was recorded digitally (both *p* < 0.001; Table 2; Figure 4). 

**Figure 5:**
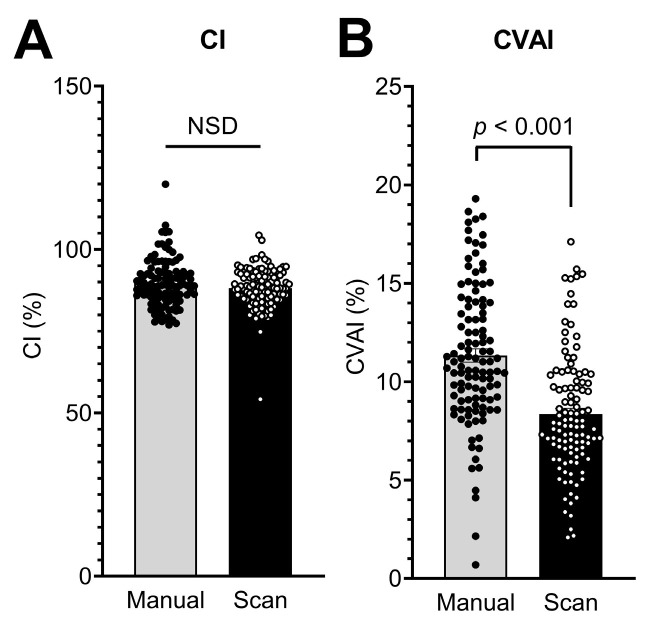
Calculation of (**A**) the cranial index (CI) and (**B**) the cranial vault symmetry index (CVAI) based on data assessed manually and by 3D photographs (scan) in 111 infants. Bars represent means ± SEM.

As in the manual measurements, CI and CVAI were calculated from the measured parameters. CI by digital measurements had a mean value of 88.19 ± 6.16%, and CVAI had a mean value of 8.36 ± 3.07%. The calculated Cis of the two measuring methods did not show significant differences (*p* = 0.343; Table 2). On the other hand, CVAIs calculated from digital measurements showed a 0.74-fold decrease compared to CVAIs from manual measurements (*p* < 0.001; Table 2; Figure 5A,B).

## 4. Discussion

This study aimed to evaluate digital 3D photographic imaging as a sufficiently precise and reproducible alternative to manual anthropometric measurements in children with DP. Direct anthropometric measuring, using calipers and a tape measure, is the most commonly used method for acquiring cranial parameters [4,19,22,23]. However, by requiring good patient cooperation, an experienced examiner, and ideally standardized head positioning, it is only possible to reach satisfactory measurement results in some clinical settings [19].

Computed tomography (CT) has been shown to be an excellent measuring method for head deformities regarding accuracy, reproducibility, as well as inter- and intrarater reliability [23,31]. However, due to the radiation exposure, it is not considered the preferred measurement method [11,23]. In addition to radiation, the need for the sedation of the infants would increase the risk for the examinee even further, again highlighting the insufficiency of CT as a primary diagnostic tool in this patient group. It should therefore be reserved for cases where synostosis could not be ruled out otherwise [11,16,21,32]. Consequently, a radiation-free alternative to CT was needed and found in 3D digital photography, as Mendonca et al. could show that both methods produce interchangeable measurement results [31,32]. In performing their manual and digital measurements on mannequins and, therefore, equal standardized settings, Weinberg et al. also confirmed that 3D digital photography is sufficiently concordant, accurate, and precise compared with CT, which makes it consequently suitable for clinical use [33]. 

Schaaf et al. and Wilbrand et al. demanded that a reliable, standardized photographic assessment setup should be established for measurements [19,29]. To fulfill this requirement with our method, a fixed toddler chair was positioned in the center of a five-camera arrangement with consistent camera distances and multi-flash lighting installations for each examination. Here, the examinee was placed sitting and, with the help of a stuffed animal in front of him or her, distracted during the short acquisition time to keep the head still and in the correct position facing forward. Additionally, standard head positioning could be reached because of the digital 3D nature of this method, and the remaining minor deviations of the standardized head position could be corrected digitally in the software by bringing the 3D model of the child´s head in the correct orientation. This provides, in summary, uniform, reliable, and reproducible measurement conditions.

A major advantage of 3D photography is the ability to acquire high-resolution surface data at relatively fast speeds (<400 milliseconds), which is particularly important when working with infants [29,32,33,34,35]. It has even been shown that 3D digital photography allows for a single trained observer to repeatedly localize anthropometric landmarks from 3D captures with a very high precision [33]. This eliminates the need for an experienced examiner, which has been required for the manual method [19,26]. 

With the development of advanced photogrammetry systems and software over the past years, a slight but significant superiority of 3D digital measurements over direct manual anthropometry could be determined [18,29,31,36]. That superiority is based on the more straightforward clinical applicability, the easier detection of landmarks without patient movements, repeatable measurements without patient discomfort, less dependence on the examiner, and better interrater reliability [29,35,36,37]. 

In line with the recent literature regarding the precision of our method, we have found that direct anthropometry generated lower values for cranial vault asymmetry parameters compared to 3D digital photography [29,31,38]. Values of RD and LD were recorded in the 3D photographs with 14.15 ± 0.90 cm and 14.37 ± 0.89 cm, respectively. The same parameters in manual anthropometry only showed values of 13.64 ± 1.06 cm and 13.41 ± 1.12 cm, respectively. In manual head circumference measurements using the tape measure, the aberration between the two methodologies could be attributed to the difficulty in finding the one horizontal plane with the biggest circumference in direct anthropometry, which can be easily detected by the digital measurement technique. Herein, 3D photography showed a mean value of 43.24 ± 1.90 cm, whereas manual measurements measured a mean circumference of only 42.76 ± 1.87 cm. A consequential error can occur, as all CI and CVAI parameters should be measured in the plane of the greatest circumference, adding up to an even larger measurement error. 

So, first, it is required to find the correct horizontal plane with the calipers, which depends heavily on the examiner’s sense of proportion and experience [19]. Second, staying in that horizontal plane and performing all measurements is challenging, while handling calipers on the moving infant’s head can be another act of defiance on its own [27,35,38,39]. Further reasons were also discussed in the literature: the required care to accurately locate bony landmarks and to prevent inaccurate measurements due to soft tissue displacement if the calipers are pressed too firmly against the infant’s head [27]. Moreover, as auxiliary lines are unavailable in direct anthropometry, RD and LD were taken roughly 30 degrees off the anteroposterior diameter, representing another limitation, which is of no concern in 3D digital photography, as the software can generate those measurements precisely at 30 degrees in the correct measurement plane [26,38].

In our direct anthropometric measurements, the sum of those potential errors was apparent, as the values of head length, head width, RD, and LD differed by more than 5 mm on average compared to the digital values (Figure 2 and Figure 3), confirming the advantages of 3D digital photography over manual caliper measurements. Our data have shown that, in three cases, 3D digital photography generated CVAI values. indicating that infants had normal head physiologies with values below 3.5%. However, manual measurements indicated values above this threshold, which would have led to helmet treatment in those patients.

Overall, direct anthropometry measurements in our study generated a mean CVAI value that was 2.97 percent higher compared to that generated by our digital method, and considering that a CVAI of 3.5% already represents pathologic cranial symmetry conditions, this error could lead to the overtreatment of infants simply by choosing the direct anthropometry method [30,40,41]. The same finding has also been described by Kato et al., who compared 2D and 3D evaluations of cranial asymmetry, again emphasizing the limitations of 2D methods for measuring 3D parameters of the infant’s skull [27].

Besides the benefits of clinical applicability, examination time, standardization, reproducibility, and accuracy, particularly noteworthy advantages of 3D digital photography over manual caliper measurements are the objectivity and archivability of the acquired photographs, where repeated measurements can be applied [29]. This allows for following the dynamic therapeutic progress accurately. In Germany, for instance, manually acquired measurements using calipers are not billable to health insurance companies. With 3D photographs as proper documentation and the transferability of those measurements, this instance could be changed, contributing to lower costs for the parents and, therefore, reducing the hurdle of having their children checked for cranial asymmetries in the first place.

Our study has some limitations since we did not have CT scans from the children’s heads. With these data as a reference point, it would have been possible to compare the measured values with the actual anatomical situation of the skull. Another limiting factor of this study was that measuring points were added to the software after the acquisition. As a result, examiners’ landmarking may have slightly differed from one another, although there is less variation compared to the manual method. Regarding future prospects, an ongoing study is in progress on orthopedic helmet fitting in children with deformed plagiocephalus. More than 400 patients were included. Symmetry measurements were performed with 3D digital photography, and the results were evaluated.

In conclusion, objectivity, a standardized setting, faster examination times, and more accurate values, together with putting less strain on all parties involved, provide solid evidence for implementing 3D digital photography as the primary diagnostic tool for evaluating deformational plagiocephaly in children. Nevertheless, because they are cheap and sufficiently easy to use, measuring calipers should not be discarded and could still be a backup alternative to 3D photography [29].

## 5. Conclusions

Our acquired data show significant differences between direct anthropometry and 3D digital photography. Using the manual method, CVAI calculations overestimated asymmetry, and cranial vault symmetry parameters were measured too low, contributing to a misrepresentation of the actual anatomical situation. Considering consequential errors in therapy choices, we suggest implementing 3D photography as the primary tool for diagnosing deformational plagiocephaly and positional head deformations. This method hardly depends on the examiner’s experience or the infants’ cooperation and offers an objective, standardized, noninvasive, and quick way of obtaining reliable data.

## Figures and Tables

**Table 1:** Clinicopathological characteristics of the patient cohort.

Category	MV ± SD (Range)	Total (*n* = 111)
Gender:		
Female	-	41 (36.9%)
Male	-	70 (63.1%)
Age at start of therapy (months)	5.59 ± 1.76 (3 to 12)	-
Diagnosis:		
Plagiocephalus	-	103 (92.8%)
Brachycephalus	-	8 (7.2%)
Skull base involvement:		
Yes	-	101 (91%)
No	-	10 (9%)
Head side:		
right accented	-	65 (58.6%)
left accented	-	46 (41.4%)
Therapy duration (days)	150.78 ± 39.11 (58 to 266)	-
Type of birth:		
Spontaneous birth	-	61 (55%)
Spontaneous delivery with suction cup	-	7 (6.3%)
Cesarean section	-	39 (35.1%)
Emergency section	-	4 (3.6%)
Twins:		
Yes	-	11 (9.9%)
No	-	100 (90.1%)

MV = mean value; SD = standard deviation.

**Table 2:** Analysis of the parameters using a manual and scan-based measurement method.

*n* = 111	Measurement Method MV (SD)			Fold of Manual
	Manual	Scan		
Circumference (cm)	42.76 (1.87)	43.24 (1.90)	*p* = 0.046	1.01
Length (cm)	13.53 (0.79)	14.33 (0.72)	*p* < 0.001	1.06
Width (cm)	12.10 (0.77)	12.67 (0.68)	*p* < 0.001	1.05
RD (cm)	13.64 (1.06)	14.15 (0.90)	*p* = 0.002	1.04
LD (cm)	13.41 (1.12)	14.37 (0.89)	*p* < 0.001	1.07
Diagonal difference (cm)	1.44 (0.41)	1.13 (0.39)	*p* < 0.001	0.78
GTR (cm)	11.26 (0.94)	10.77 (0.75)	*p* < 0.001	0.96
GTL (cm)	11.35 (0.93)	10.70 (0.68)	*p* < 0.001	0.94
CI (%)	89.67 (7.28)	88.19 (6.16)	*p* = 0.343	0.98
CVAI (%)	11.33 (3.56)	8.36 (3.07)	*p* < 0.001	0.74

MV = mean value; SD = standard deviation; RD = 30-degree diagonal right; LD = 30-degree diagonal left; GTR = glabella-tragus distance right; GTL = glabella-tragus distance left; CI = Cranial Index; CVAI = Cranial Vault Asymmetry Index.

## Data Availability

Data can be obtained from the scientists that conducted the work independently from the industry on request. The data are not stored on publicly available servers.

## Supplementary Materials

The following supporting information can be downloaded at: https://www.mdpi.com/article/10.3390/diagnostics13101707/s1, Figure S1: Setup and adjustment of the Vectra M5 360 camera system with a fixed toddler chair.
